# A Rare Case of Iliopsoas Abscess Extending to Scarpa’s Triangle With Gluteal Fistulization

**DOI:** 10.7759/cureus.91473

**Published:** 2025-09-02

**Authors:** Tudor Negruti, Marian Botoncea, Catalin D Cosma, Dragos Molnar, Calin Molnar

**Affiliations:** 1 General Surgery, County Emergency Clinical Hospital of Târgu Mureș, Târgu Mureș, ROU; 2 General Surgery, Spitalul Clinic Județean de Urgență Târgu Mureș, Târgu Mureș, ROU; 3 General Surgery, George Emil Palade University of Medicine, Pharmacy, Sciences and Technology, Târgu Mureș, ROU; 4 Surgery, George Emil Palade University of Medicine, Pharmacy, Sciences and Technology, Târgu Mureș, ROU

**Keywords:** case report, fistulization, iliopsoas abscess, retroperitoneal infection, scarpa’s triangle

## Abstract

Iliopsoas abscess (IPA) is a rare and potentially life-threatening condition, often presenting with nonspecific symptoms that delay diagnosis. Secondary IPA, typically resulting from contiguous spread of infection, can occasionally demonstrate atypical anatomical extension.

We report the case of a 64-year-old female patient with a history of right nephrectomy, type 2 diabetes mellitus, and hypertension, presenting with a progressively enlarging mass in the right thigh. Imaging revealed a large multiloculated retroperitoneal abscess originating from the iliacus muscle and extending through the psoas to the right Scarpa's triangle and gluteal region, with potential active fistulization. The patient underwent extensive surgical drainage, debridement, fistulectomy, and dual-site drainage, followed by targeted antibiotic therapy. Postoperative recovery was favorable.

This case highlights a rare and complex anatomical presentation of recurrent IPA. The extensive spread beyond classical retroperitoneal boundaries into Scarpa’s triangle and the gluteal soft tissues underscores the importance of early imaging, surgical management, and multidisciplinary care in complex abscesses.

A high index of suspicion, appropriate imaging, and individualized surgical intervention are essential for managing complex IPA. Atypical presentations such as this case warrant prompt recognition to prevent morbidity and ensure favorable outcomes.

## Introduction

Iliopsoas abscess (IPA) is a rare but potentially life-threatening condition, with an estimated prevalence of 0.4 per 100,000 individuals annually in high-income countries like the United Kingdom [1]. The disorder is defined by the accumulation of pus within the iliopsoas compartment, which may arise via hematogenous spread from a remote infected site (primary IPA) or through direct extension from adjacent structures (secondary IPA) [1,2]. The iliopsoas muscle originates from the lateral borders of the T12 to L5 vertebrae and inserts into the lesser trochanter of the femur. Owing to its proximity to retroperitoneal and intra-abdominal organs, including the kidneys, ureters, appendix, pancreas, and bowel, it is susceptible to infection from a variety of sources, including gastrointestinal, genitourinary, and spinal pathologies.

Historically, Mynter described the condition as “psoitis” in 1881. Before the advent of antibiotics, IPA was a common complication of spinal tuberculosis. The decline in tuberculosis incidence in high-income nations has shifted the epidemiology of IPA. Currently, most cases are secondary to inflammatory or infectious processes, such as Crohn’s disease, diverticulitis, appendicitis, or spinal osteomyelitis [1]. Primary IPA occurs predominantly in children and immunocompromised individuals, especially those with HIV. It is more prevalent in Asia and Africa, accounting for more than 90% of cases in these regions, compared with 18.7% in Europe [2].

The clinical presentation of IPA is varied and often nonspecific. The classic triad of fever, back pain, and limp is well recognized but is present in only 30% of patients [2]. The ambiguous nature of symptoms such as indistinct stomach pain or groin discomfort from lumbar nerve involvement frequently leads to diagnostic delays. This subsequently increases the risk of complications such as abscess rupture, sepsis, and atypical fistula formation. Mortality rates range from 5% to 15%, and recurrence rates approach 16% despite appropriate intervention [1].

Management typically involves antibiotic medication in combination with image-guided or surgical drainage. Nonetheless, complex or recurrent presentations, particularly those affecting adjacent fascial planes, such as Scarpa’s triangle, or those progressing to remote fistulization, as illustrated in our case, are infrequently reported and pose unique diagnostic and therapeutic challenges. This case report seeks to emphasize an unusual and intricate presentation, highlighting the necessity for increased clinical vigilance and tailored therapeutic options in IPA.

Recent literature has expanded understanding of IPA epidemiology, pathogenesis, and clinical spectrum. Tabrizian et al. [3] reported that open surgical drainage remains essential for complex, multiloculated, or recurrent IPA. Wong et al. [4] emphasized the role of rapid imaging-based diagnosis in reducing morbidity. López et al. [5], in a review of 124 cases, identified Escherichia coli and Staphylococcus aureus as the most common pathogens in secondary and primary IPA, respectively. Ricci et al. [6] highlighted geographic variations in IPA origin, while Li et al. [7] described rare causative organisms such as Streptococcus dysgalactiae in immunocompromised patients. These findings reinforce the need for tailored management strategies based on anatomical complexity, microbiological profile, and host status.

## Case presentation

A 64-year-old female patient with a history of right nephrectomy (1993), laparoscopic cholecystectomy (2020), type 2 diabetes mellitus, and grade 2 arterial hypertension was admitted electively with a tumoral-like mass in the right thigh of approximately 1 month’s duration, which had progressively enlarged. During the clinical examination, the mass was situated in the right femoral triangle (Scarpa’s triangle) and was initially suspected to be a lymphocele.

On examination, the abdomen demonstrated symmetric respiratory movement in the xipho-pubic plane. The umbilicus was normal in shape and position, with well-healed postoperative scars along the midline xipho-pubic region and right flank. In the right inguino-crural region, a pseudotumoral, non-tender, fluctuant mass measuring approximately 6 × 4 cm was palpated. The remainder of the abdomen was supple, elastic, and non-tender on both superficial and deep palpation, with normal bowel sounds.

**Figure 1:**
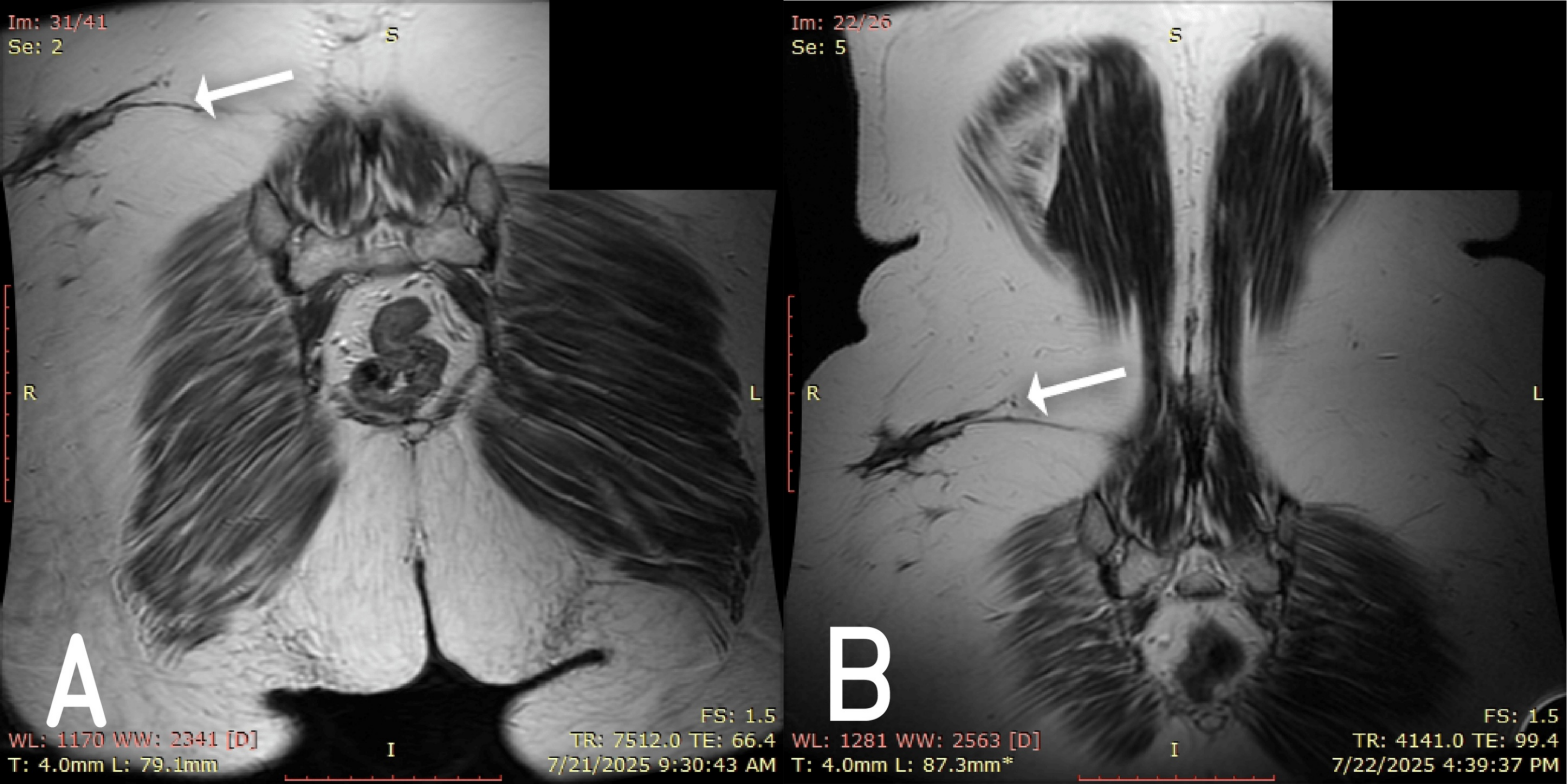
Preoperative pelvic MRI showing fluid accumulation with fistulization to the retroperitoneum (white arrows).

Pelvic MRI revealed a multilobulated fluid accumulation measuring approximately 21 × 10 × 7.5 cm (Figure 1). The collection originated paravertebrally on the right, following the course of the right iliacus muscle, extending into the pelvic fossa and along the ipsilateral psoas major muscle to the anteromedial surface of the right thigh. Surrounding soft tissues exhibited diffuse swelling, and the collection was in direct contact with the right external iliac vascular axis.

**Figure 2:**
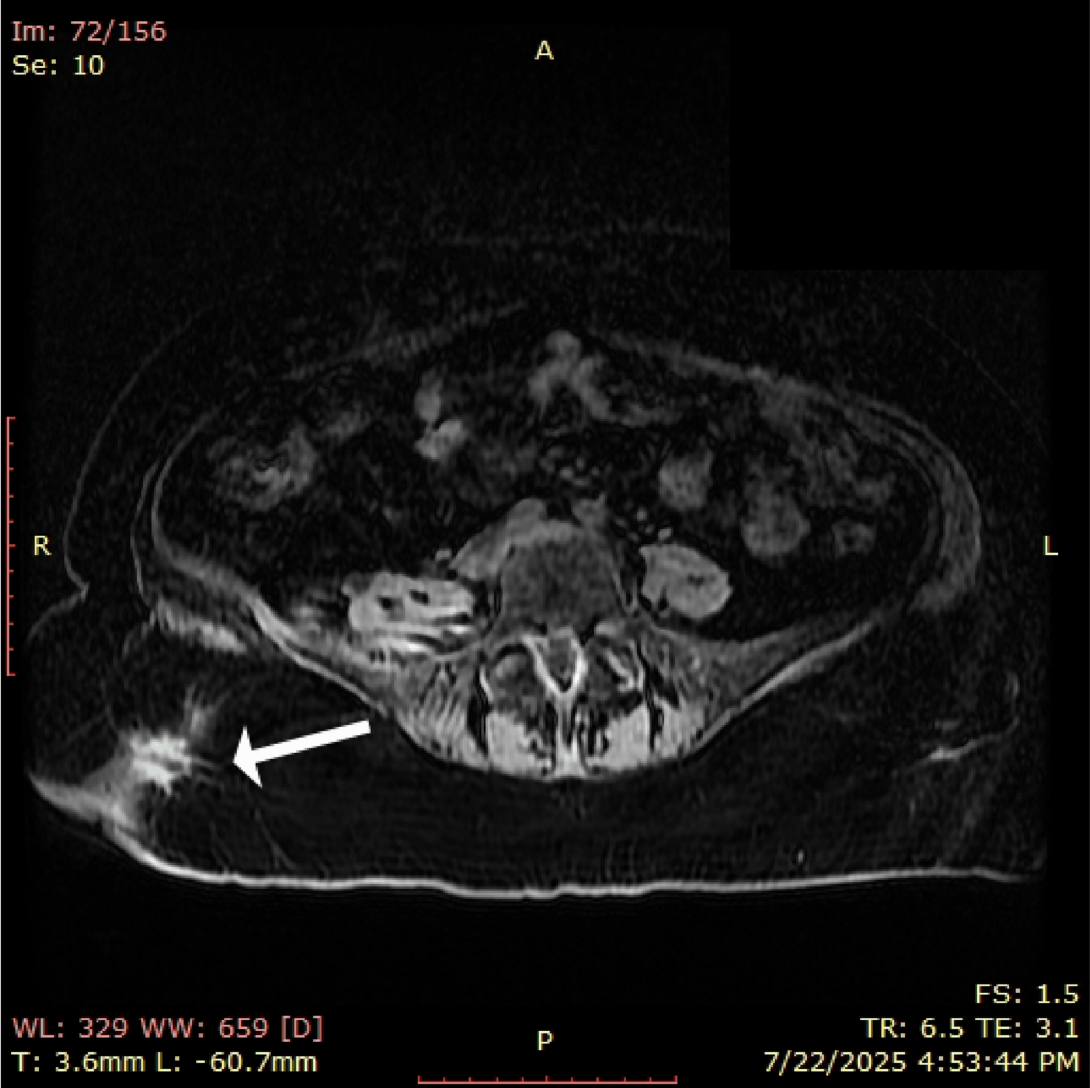
Preoperative lumbar spine MRI showing multiloculated paravertebral collection (white arrow).

Lumbar spine MRI confirmed a multiloculated paravertebral collection arising from the right iliacus muscle near the L1 vertebra (Figure 2). The collection exhibited a gadolinium-enhancing wall of varied thickness (1-6 mm) and communicated with a tract leading to the subcutaneous lumbogluteal plane. Two gadolinium-enhanced active fistulous tracts extended to the cutaneous level in the right gluteal region, measuring approximately 9 mm and 11 mm in diameter.

**Figure 3:**
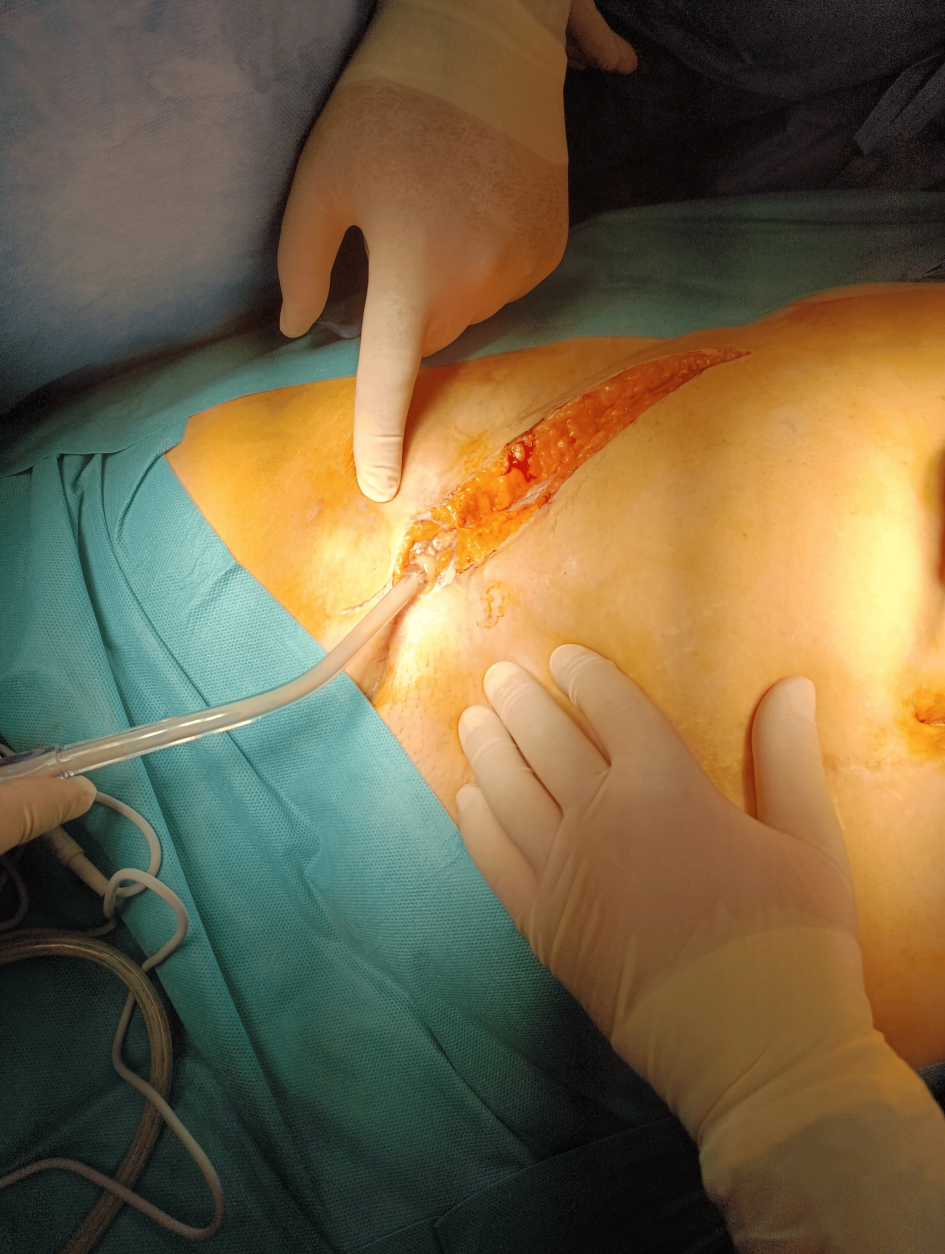
Inguino-crural incision with pus drainage from the tract in Scarpa’s triangle.

**Figure 4:**
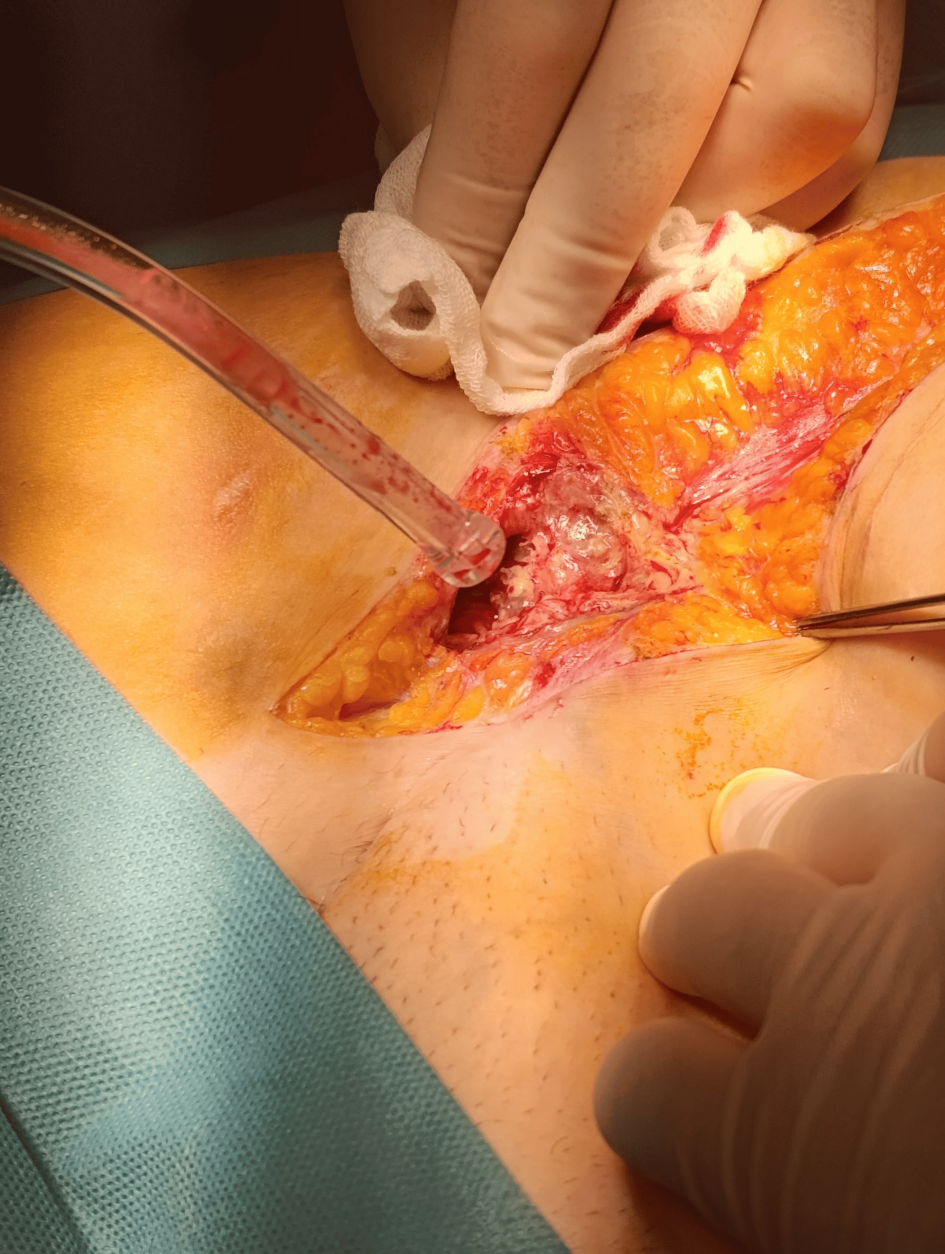
Retroperitoneal fusiform collection along the inguino-crural path.

**Figure 5:**
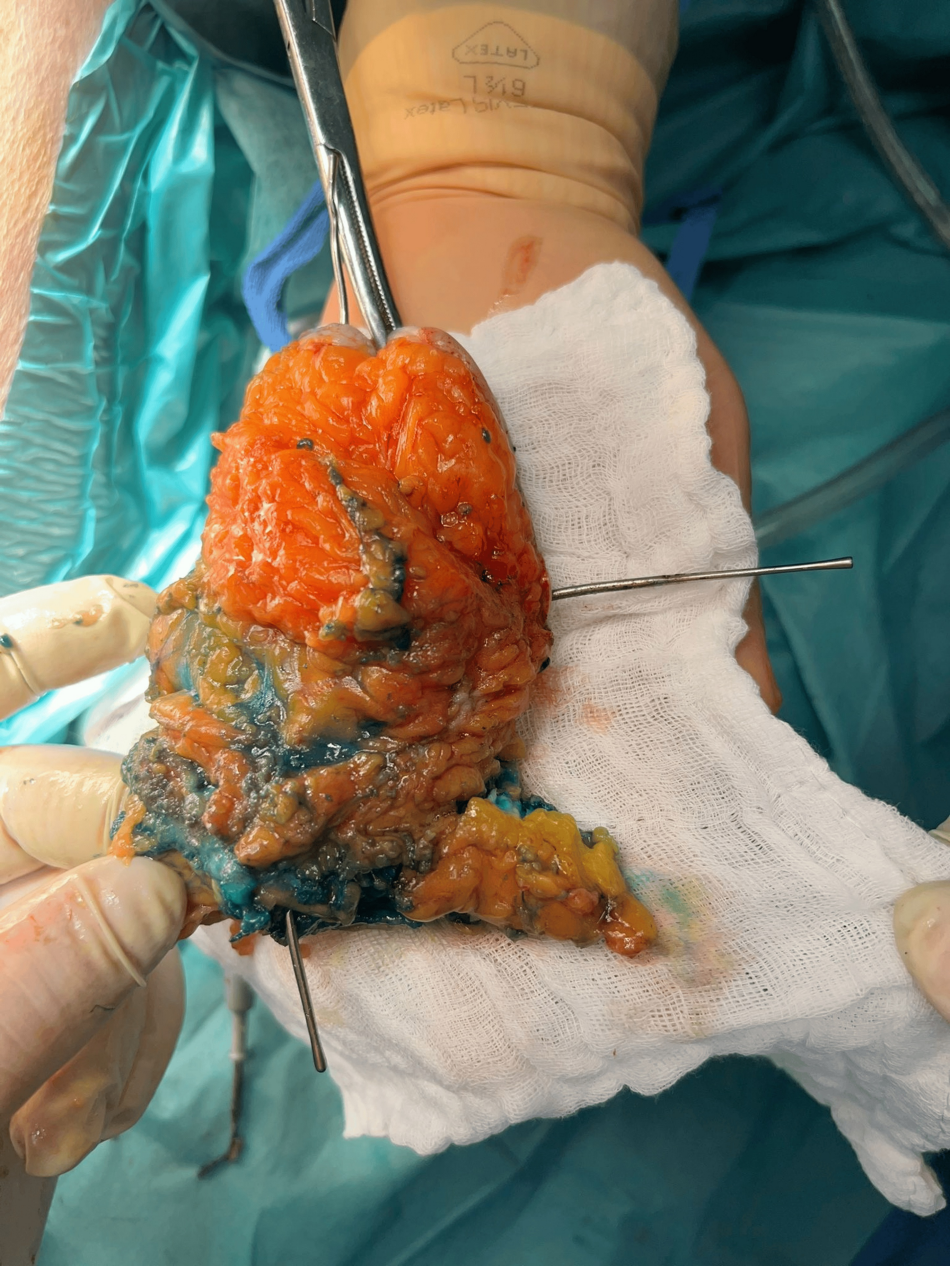
Gluteal fistulectomy with intraoperative methylene blue fistulography (secondary tract marked with the stylet).

**Figure 6:**
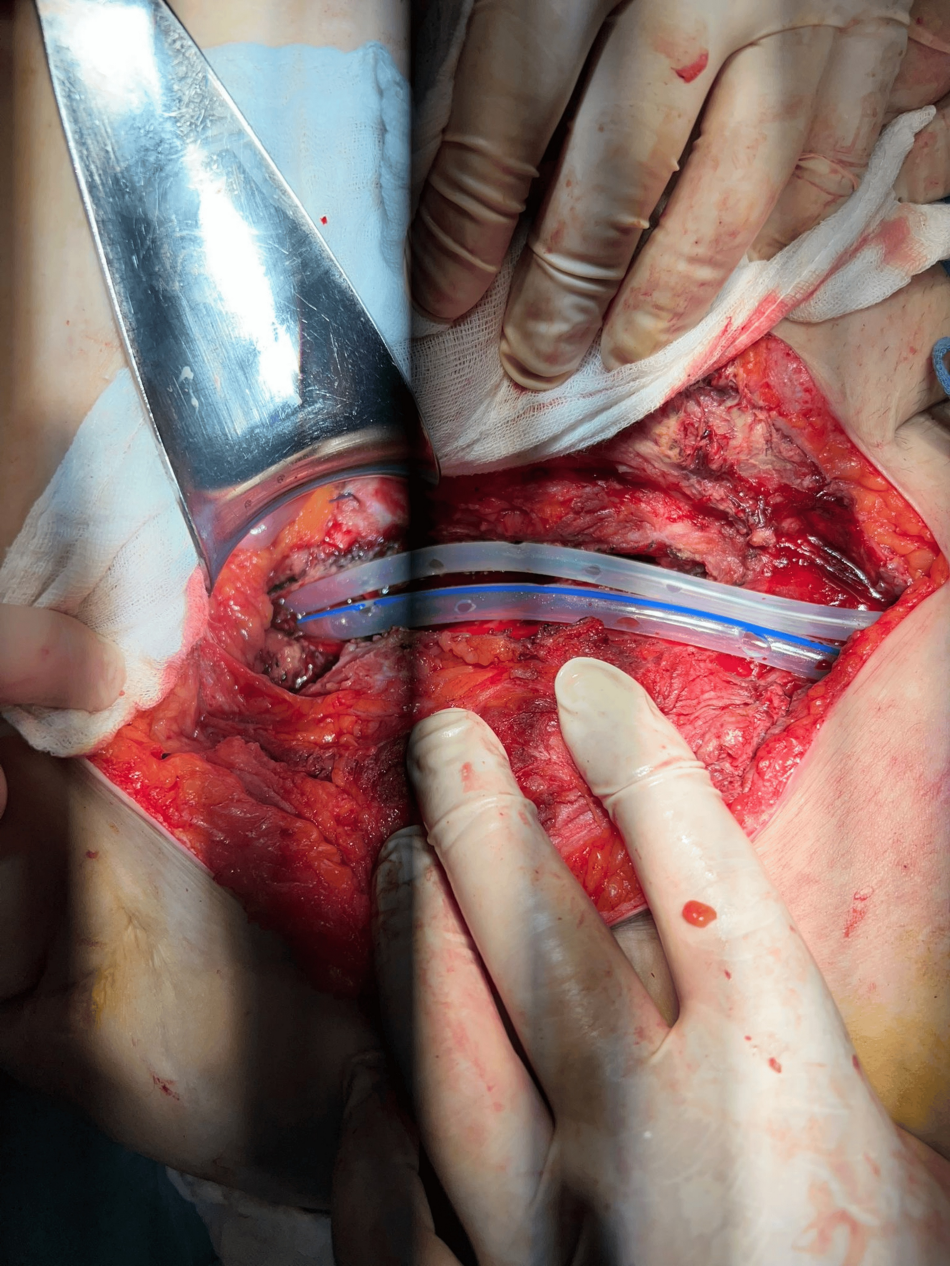
Placement of drains along the retroperitoneal inguino-crural tract.

**Figure 7:**
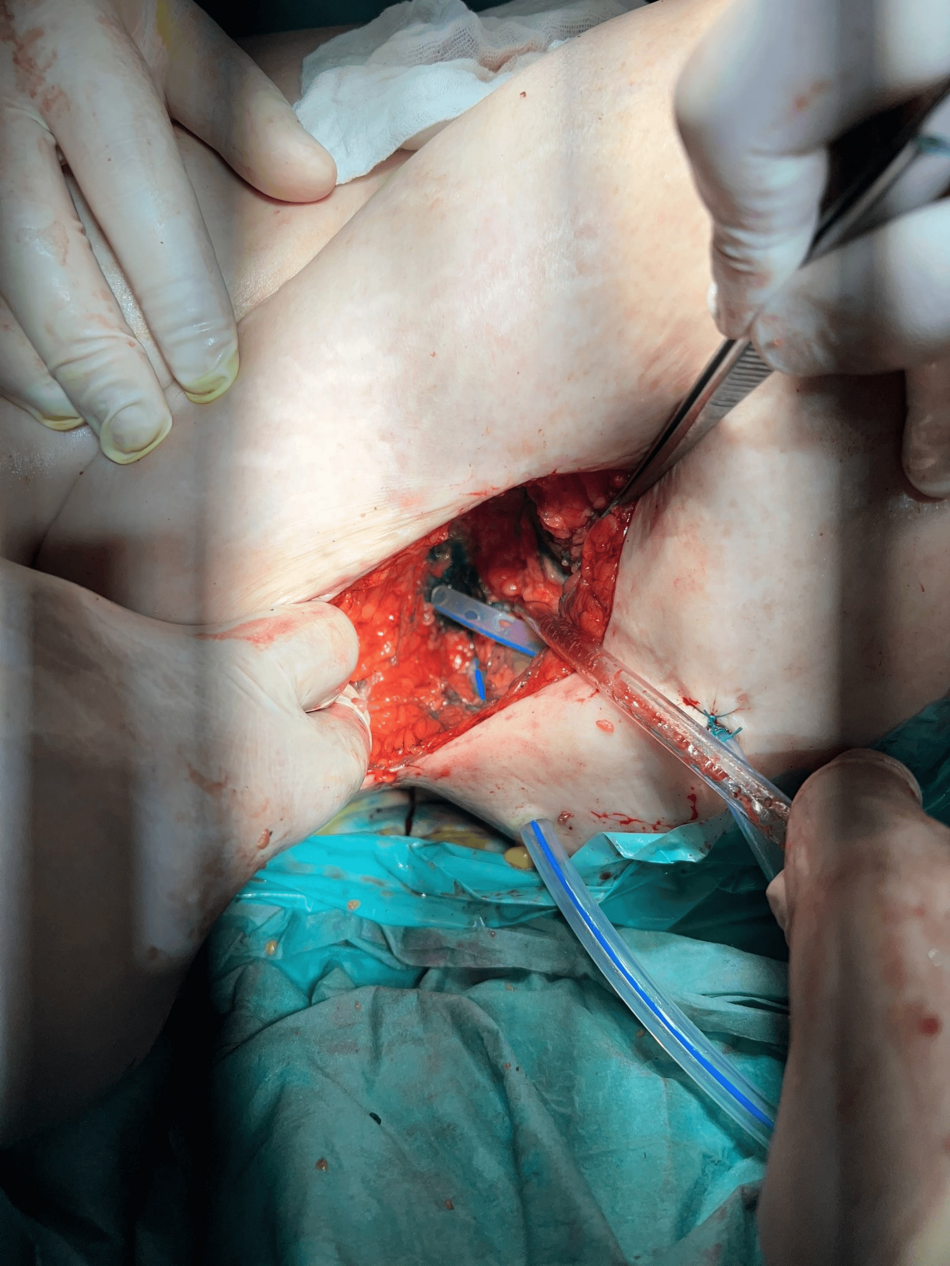
Placement of drains in the gluteal region.

Following preoperative optimization, surgery was performed on the third day of admission. Intraoperative findings included a retroperitoneal abscess extending into the right inguino-crural region (Figures 3, 4), fistulization to the right gluteal area, mesh granuloma, and necrosis of the right iliacus muscle. Procedures performed included evacuation and lavage of the retroperitoneal abscess, chromographic fistulography (Figure 5), fistulectomy of the right gluteal region, excision of suture granuloma, necrectomy of the right iliacus muscle, and placement of double retroperitoneal (Figure 6) and gluteal drainage (Figure 7), followed by skin closure.

Intraoperatively, a purulent material was collected for bacteriological examination; the results showed frequent leukocytes (sterile pus).

**Figure 8:**
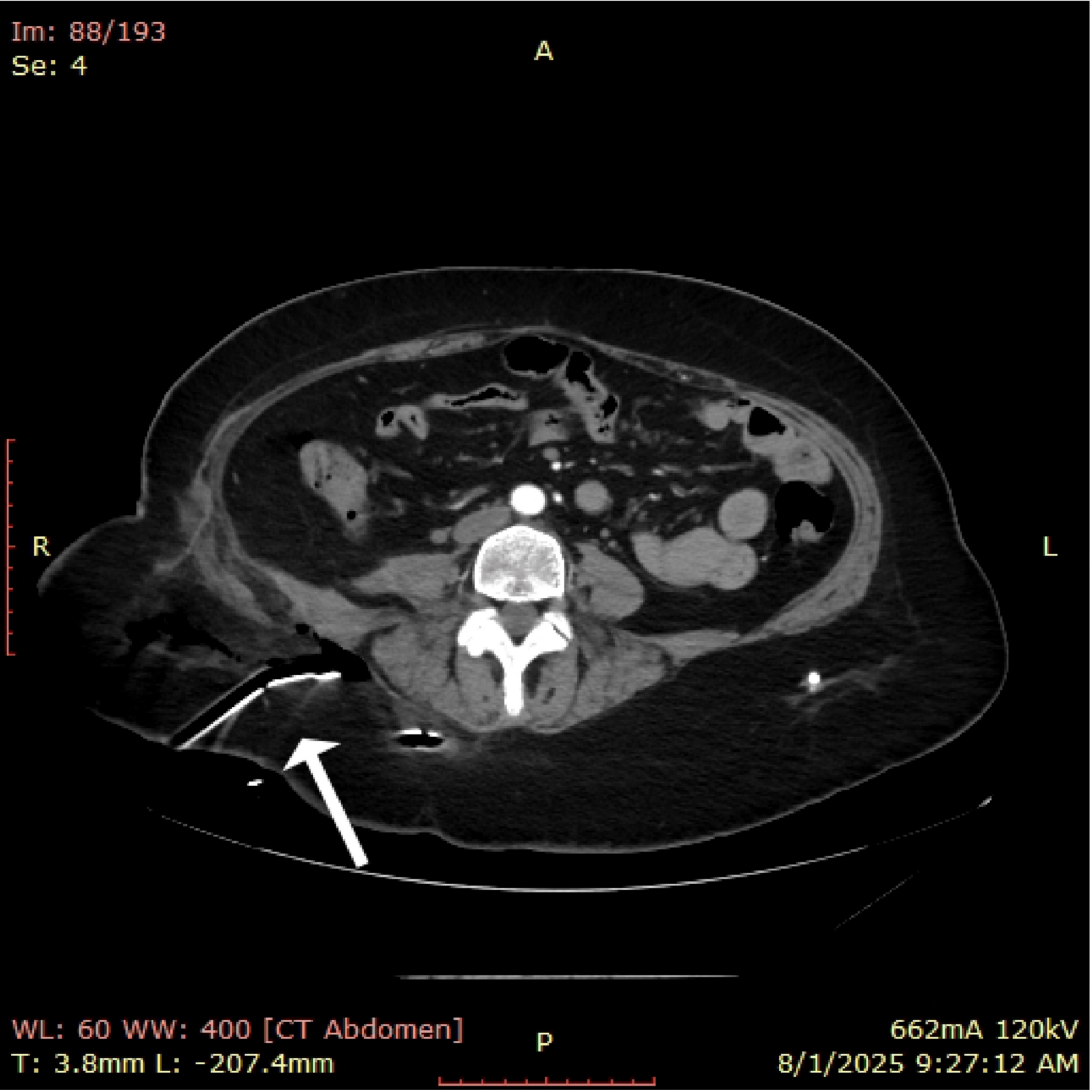
Postoperative CT scan showing drains in situ (white arrow).

The postoperative recovery was positive (Figure 8). The patient received empiric antibiotic therapy consisting of intravenous meropenem 1 g administered three times daily and teicoplanin 400 mg twice on day 1, then 400 mg daily thereafter, per infectious diseases consultation. Renal function was meticulously observed, with dosage adjustments based on creatinine clearance.

By postoperative day 12, the patient was discharged in satisfactory general condition. She was afebrile, alert, and cooperative, with a Glasgow Coma Scale score of 15, stable vital signs, normal bowel function, and spontaneous urination. All surgical drains had been removed. The patient received empiric antibiotic therapy for 12 days, as recommended by the infectious disease specialist. The drainage tubes utilized were simple polyethylene catheters. The initial output from each drain was approximately 50 mL. Beginning on postoperative day 5, the drains were progressively and alternately shortened. Both drains were simultaneously removed on day 11.

## Discussion

IPA is a rare but potentially life-threatening condition that often presents with ambiguous, nonspecific symptoms, leading to diagnostic delays. The classic triad of fever, back pain, and limp is present in only approximately 30% of cases; many patients instead exhibit mild, nonspecific symptoms such as malaise, hip pain, or lower abdominal discomfort [1-4]. The unusual location of the abscess in this case, with extension into Scarpa’s triangle and fistulization to the gluteal region, emphasizes the diverse presentations of IPA and the diagnostic challenges it presents.

IPA is classified as primary or secondary according to its origin. Primary IPA, which accounts for approximately 30% of cases, results from hematogenous spread without an identifiable adjacent infectious source. It is more common in immunocompromised individuals and pediatric patients [2,4]. Secondary IPA arises from contiguous spread from adjacent structures and constitutes the majority of cases, particularly in Western countries [2]. Ricci et al. found that more than 90% of IPA cases in Asia and Africa are primary, compared with only 18.7% in Europe [6].

The current case represents the secondary subtype, most likely arising due to a chronic, undrained retroperitoneal infection. The patient's history of nephrectomy and cholecystectomy may have altered regional anatomy (the local anatomy was significantly altered due to necrosis of the quadratus lumborum, iliopsoas, internal oblique, and transversus abdominis muscles) and local immune responses, while comorbidities such as type 2 diabetes mellitus increased her vulnerability. Diabetes is a recognized risk factor for both primary and secondary IPA because of impaired neutrophil function and microvascular changes [8].

Anatomically, the psoas and iliacus muscles connect the abdomen to the lower limb, making them vulnerable to infection from multiple sources. The iliopsoas compartment lies adjacent to the kidneys, ureters, pancreas, colon, vertebral bodies, and iliac arteries, facilitating direct spread from these organs [1,4,5]. In this patient, the abscess extended from the right iliacus muscle to the ipsilateral psoas, following fascial planes to Scarpa’s triangle and into the subcutaneous tissue of the gluteal region. While IPA typically remains confined to the retroperitoneal space, fistulization to the skin, particularly to remote areas such as the gluteal region, is exceptionally rare and indicates a chronic infection with significant necrosis [9].

Radiologic imaging was essential for diagnosis and surgical planning. MRI provided high-resolution delineation of the abscess and fistulous tracts. Although CT remains the gold standard for IPA diagnosis because of its near-100% sensitivity, MRI offers superior soft tissue contrast, which was particularly important in this case given the extension beyond the pelvic cavity [1,3,5]. Modern imaging also facilitates detection of complex, multiloculated abscesses that may be missed on physical examination or ultrasonography [10].

Microbiological diagnosis and targeted antibiotic therapy are central to IPA management. Secondary IPA pathogens often include Escherichia coli, Staphylococcus aureus, Bacteroides species, and Enterococcus species, depending on the source of infection [2,4,6]. This patient received empiric broad-spectrum treatment with meropenem and teicoplanin, consistent with guidelines for suspected polymicrobial abscesses of gastrointestinal or genitourinary origin. Although final culture results were pending at discharge, intraoperative findings of necrotic iliacus muscle and granulation tissue suggested a polymicrobial process, a common feature of secondary IPA [2,6].

The case also demonstrates the role of aggressive surgical intervention in complex IPA. While percutaneous drainage is appropriate for selected unilocular abscesses smaller than 3.5 cm in diameter, large, multiloculated, or fistulized abscesses require open surgical drainage [1,5,7]. In this patient, extensive debridement, fistulectomy, and placement of dual drains were essential for infection control. The favorable postoperative course supports the need for individualized surgical intervention when abscess anatomy precludes less invasive methods.

Recurrence of IPA can occur in up to 15.8% of cases, particularly when drainage is incomplete or the source infection is left untreated [1]. Risk is higher in immunocompromised patients, with delayed diagnosis, or in those with complex abscess morphology. In this patient, the chronicity and extent of the infection suggest a longstanding, inadequately treated process, either overlooked or misdiagnosed in prior evaluations. Long-term follow-up with imaging surveillance and assessment of inflammatory markers is recommended even after apparent clinical resolution.

The differential diagnosis of lower abdomen, hip, or flank pain is broad. The psoas muscle’s proximity to multiple abdominal and pelvic structures means IPA can mimic conditions such as appendicitis, diverticulitis, septic arthritis of the hip, vertebral osteomyelitis, sacroiliitis, and retroperitoneal malignancy [2,4]. Musculoskeletal and neurological disorders, including lumbar radiculopathy, may also be considered, particularly when pain radiates to the leg [2,4]. In this case, the extension into Scarpa’s triangle and the gluteal region may have obscured the retroperitoneal source, potentially leading to an initial misdiagnosis such as superficial lymphocele.

Physical examination findings in IPA are variable. The psoas sign, pain with passive hip extension, occurs in only a minority of cases and has low sensitivity [7]. Hip flexion deformity and guarding are more common, as patients instinctively reduce tension on the inflamed muscle [8]. In this patient, inflammation and mass effect extended into the thigh and gluteal region, producing a palpable mass, an atypical presentation more suggestive of superficial infection or femoral hernia. Only advanced imaging confirmed the diagnosis, emphasizing the importance of early comprehensive radiological evaluation in ambiguous cases.

The microbiological spectrum of IPA varies with geography and cause. Staphylococcus aureus predominates in primary IPA, whereas Escherichia coli is more common in secondary IPA linked to urinary or gastrointestinal sources [2,4]. Mixed anaerobic and aerobic organisms are frequent in secondary disease, and methicillin-resistant Staphylococcus aureus or fungal pathogens may occur in immunocompromised patients [2,4,6]. At the time of release, the culture results for our patient were still pending; however, the empiric administration of meropenem and teicoplanin effectively addressed both gram-positive and gram-negative bacteria, in accordance with best-practice guidelines [1,2].

Surgical decision-making in complex IPA must account for abscess size, location, and systemic effects. Although percutaneous drainage is less invasive and preferred for small, localized abscesses, open surgery remains the primary approach for patients with multiloculated, necrotic, recurrent, or complicated cases, including those with fistulas or mesh granulomas [1,5,6]. In this patient, the unusual fistulization to the gluteal region, in conjunction with suture granuloma and muscle necrosis, necessitated open necrosectomy and chromographic fistulography. These measures were crucial both for eradicating infection and for preventing recurrence or chronic fistula formation.

This case also highlights the importance of multidisciplinary management. Collaboration among infectious disease specialists, radiologists, and surgeons is essential to optimize diagnostic strategies, tailor antimicrobial therapy, and guide long-term follow-up plans. In patients with a solitary kidney or reduced renal reserve, as in this case, careful monitoring of renal function during antibiotic therapy and appropriate dose adjustments are imperative.

## Conclusions

IPA is an uncommon but clinically significant condition with a high risk of morbidity and mortality, especially when diagnosis is delayed or when anatomical extension complicates the presentation. This report describes an exceptionally rare case of recurrent IPA with fistulization into Scarpa’s triangle and the gluteal region, demonstrating the complex progression of untreated or partially treated retroperitoneal infections. While percutaneous drainage is appropriate in select patients, this case reinforces the role of open surgical management for large, multiloculated, or fistulized abscesses, especially when complicated by necrosis or foreign body reaction.

Timely surgical intervention, combined with broad-spectrum antimicrobial therapy and multidisciplinary postoperative care, led to a favorable clinical outcome. This case adds to the growing body of literature emphasizing the variable presentations and challenges associated with IPA and highlights the need for personalized therapeutic strategies based on anatomical complexity and host factors.
